# Supplementary material for: Ubiquitin Ligase U-Box51 Positively Regulates Drought Stress in Potato (Solanum tuberosum L.)
Source: Int J Mol Sci. 2024 Dec 2;25(23):12961. doi: 10.3390/ijms252312961 (PMC11641713; doi:10.3390/ijms252312961)
Supplement: Supplementary file 1 [file ijms-25-12961-s001.zip › ijms-3337239-supplementary.pdf]

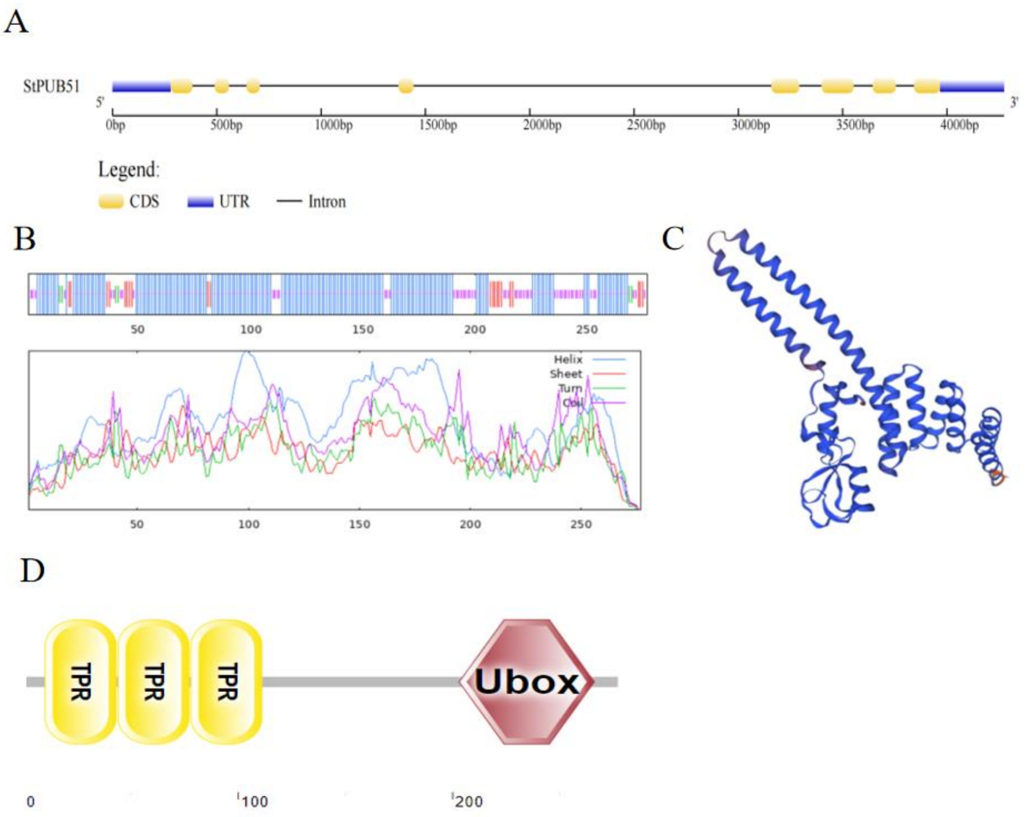

Figure S1. Bioinformatics analysis of *StPUB51*. (A) gene structure analysis.(B) Protein secondary structure analysis. (C) Protein tertiary structure analysis. (D) conserved domain analysis

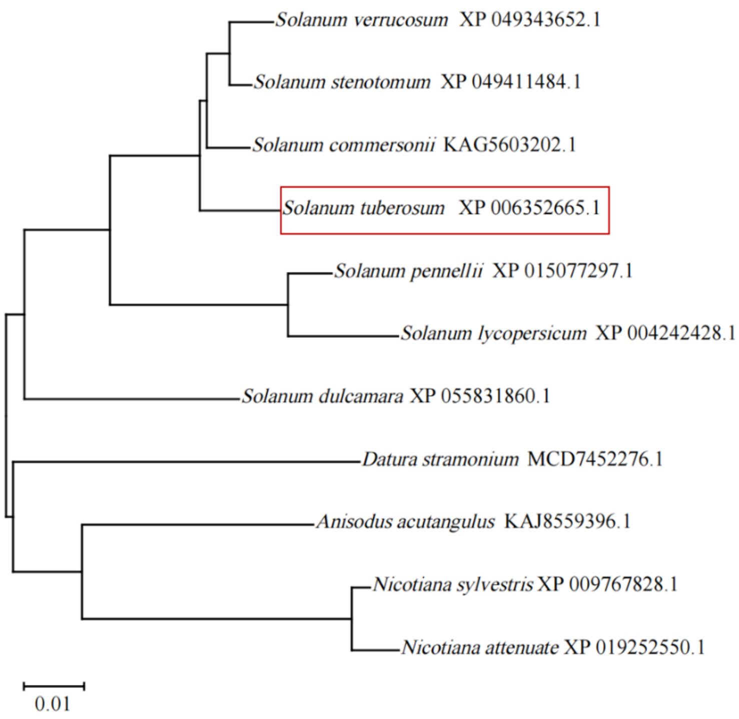

Figure S2. Phylogenetic tree of StPUB51 amino acid sequence and homologous sequences of other species

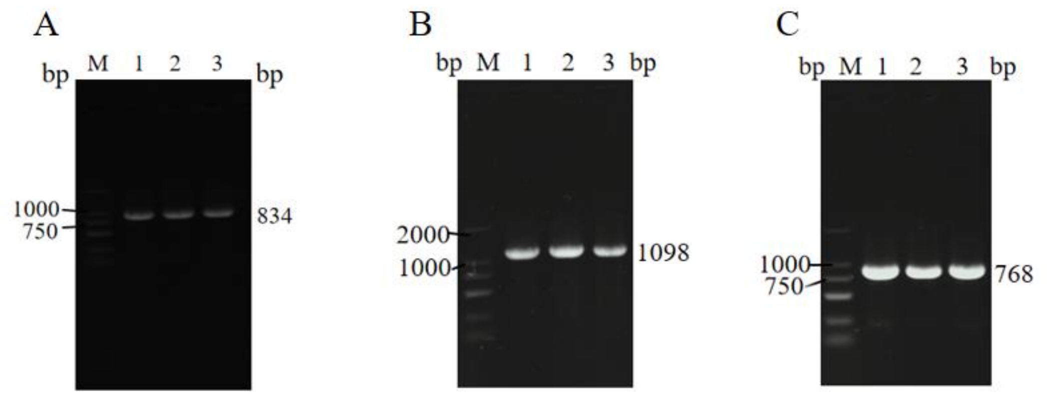

Figure S3. Cloning of *StPUB51*, *StSKP2A*, *StGATA1*
M: DNA Marker DL2000; 1: *StPUB51*, 834 bp; 2: *SKP2A*, 1098 bp; 3: *StGATA1*, 768 bp

Table S1. CDS sequences of the genes in this study

Gene	Gene ID	Coding sequence (CDS)
StPUB51（834 bp）	XM_006352603.2	ATGGCATCAATCGTGGGTTCGAAGCAAGCGGAACAACTAAAGCAAGATGGGAACCATTATTTTCAGAAGAA<br>TCGGTTTGGGGCTGCCATTGATGCTTATACCGAGGCAATTACTTTGTGCCCTAATGTTCCGATATATTGGACC<br>AATCGTGCTCTATGTCATCGGAAACGGAATGACTGGGGAAGAGTGGAGGAAGATTGCAGGAAGGCGATTCA<br>GCTCGATCATAATTCTGTAAAGGCCCACTATTATCTTGGTCTCGCATTGCTACAAAAGGAACAATATGCTGA<br>AGGTGTGAGAGAATTGGAAAAGGCATTAGACCTTGGGAGAGGTGCGAATCCAGGAAGATATATTGTTGAAG<br>AGATCTGGGAAGAGCTTGCTAAAGCAAAGTACATGGAGTGGGAGCATGAATCTACAATGCGCTCCTGGGAG<br>CTTCAGAACTTGAAAGAATCCTGTGAGTCAGCTCTCAAGGAGACCCATATGCTTGACAGTTCTCAGACAGAA<br>GGGCTCAAAGATGAAAACTCAACAGCGCTTTTGAAGCAACTGGAAGCTCTAGGTGAGGTTTTCATGAAAGC<br>TGCAGCAGATGATATTCCAACTGAGGTTCCTGATTACTTGTGTTGTAAAATTACTCTTGATATTTTTCGTGAC<br>CCTGTAATTACTCCGAGTGGGTTTACATATGAGCGGGCTGTGATCCTTGAGCATTTGCAAAAGGTGGGCAAA<br>TTTGATCCAATCACAAGAGAACAACTTTTGCCGTCCCAGTTGGTGCCAAACCTGGCCATAAAAGAATCTGTG<br>CGGGCATTTTTGGATAGGCATGGCTGGGCATACAGGATACAGTAA
StSKP2A（1098 bp）	XM_015312788.1	ATGTTAGGGGTAGAAGATTTGAGCTTGGACAAGCTGTTTGTGAACAATAGTAGCAGAGGTGGGGTTTGTAA<br>AATGGGGGGTGGGGTGATGATGATGGGAGAGTGGAAGGATATACCTATGGAGTTGTTGTTGAGGATTGCTT<br>CACTTGTGGATGATCGGACGGTGATTGTGGCTTCTGGTGTTTGTAGTGGATGGAGAGATGCAATTTCTTGGG<br>GACTCACTCGTCTTTCCCTTTCTTGGTGCAAGAGGAATATGAACAATTTAGTGCTGACACTTGCCCCCAAGTT<br>CACGAAGCTGCAGGTTCTAACTCTTAGACAAGATTTACCACAACTTGAGGATGTTGGTGTCGAGACAATTGC<br>CAATCACTGTCATGAATTGCAAGACCTTGATCTTAGCAAGAGTTTTAAGCTAACTGATCGTTCCCTTTATGCT<br>TTAGCTCACGGCTGTCCAAACCTCACAAAGCTGAATATCAGTGGGTGTTCTGCTTTCAGTGATAGCGCTGTT<br>GCATACCTAGCTGAGCATTGCCGAAACCTAAGAGTCTTAAACCTTTGTGGTTGTGTTAAAGCTGCAACTGAT<br>AAGGCATTGAAGGCTATTGGTTACTACTGTAACCGACTGCAGACTGTAAATCTTGGTTGGTGTGATAAAGTT<br>GGTGATGAAGGTGTAATGAGTTTGGCTTATGGGTGTCCTGATCTCAGAGCCCTCGATCTGTGTGGCTGTGTC

		CTTATAACAGATGAGACTGTGATTGCTTTGGCAAACAATTGCACTCACTTGAGATCCCTCGGCCTATACTTCT<br>GTCAGTACATCACAGATAGGGCAATGTACTCTCTAGCTCAGAGTCGGGTCAAGAACAAGCACGAGATGTGG<br>GCATCTATGAAGAAGCACAGGTACGAGGACGAAGGGCTTATGAATCTAAACATCAGCCAATGCACTGCTCT<br>CACACCTCCCGCCGTTCAAGCAGTATGCGAAGCATTTCCTGCTCTGCACACATGCCCTGAGCGACATTCCCT<br>CATCATTAGCGGATGCCTGAACTTAACATCAGTGCACTGCGCCTGCTCTGTCCAAGCTCACCGTGCGCGTGT<br>TCTCCATCCAGCTCACTGA
StGATA1<br>(768 bp)	XM_006355679.2	ATGAAAATGGAAGCTATGTACTCAAAAACTTGTTTTATGGTAGATGATGATATTCTTAATTTCTCTTTAGATG<br>ATGATGAAAAATATCAAACTTCTTCTTCTTCTTTTGAATCATCAAGCACCCTTGGATTTCATCAAGATGATCA<br>TAGCCCTTCATTTCCTGATTATGTAGAGGAAGAATTGGAATGGCTTTCAAATAAAGATGCATTTCCAGCAGT<br>AGAATTTGACATATTCTCTGATCATGTCCCTAATGTCATATTTGATCACCACAGCCCAAATTCAGTACTGGAA<br>AACAGCAGCAGCAACAACAACAACAACAACAATTGCAACGTAAATGTTAAGAAAAACGCGTTTACAAGCC<br>ACACCTCTAGCCTCCTACAAGTCCCCATTAACCACCCCGTTGGTGCACGGAGCAAGAGAAGGCGAAGAATC<br>GCCTTGCAATGTGACAACTCATGTGTATGGGGTAACCAAGTGAAATTCAACAACACTAGTACTAAGCAAGG<br>ACTAACATTGCTCAAAATATCAATGACTAAAGCTAAAAGAGGTACTAGCATTGGTAGGAGGTGTCAGCATT<br>GTGGGGTCGATAAGACCCCACAATGGCGTGCAGGTCCTACAGGACCTAAAACGTTGTGCAACGCTTGTGGG<br>GTCCGGTATAAGTCTGGAAGGTTGTTCCCTGAGTATCGCCCCGCGAATAGCCCTACTTTTTCAGTTGATTTAC<br>ATTCAAGTTCTCATAGGAAAGTTTTGGAGATGAGAAAGCAGAGGATTTAA

Table S2. Predicted cis-acting elements in the promoter region of the *StPUB51*

Name	Squence	Numble	Function
BoxII	CCACGTGGC	1	part of a light responsive element
ABRE	CACGTG	2	cis-acting element involved in the abscisic acid responsiveness
I-box	GATAAGGGT	1	part of a light responsive element
GA-motif	ATAGATAA	2	part of a light responsive element
MSA-like	TCCAACGGT	1	cis-acting element involved in cell cycle regulation
TATA-box	TATA	14	core promoter element around -30 of transcription start
GATA-motif	AAGATAAGATT	1	part of a light responsive element
G-box	CTTCCACGTGGCA	5	cis-acting regulatory element involved in light responsiveness
CCAAT-box	CAACGG	1	MYBHv1 binding site
CAAT-box	CAAAT	1	common cis-acting element in promoter and enhancer regions
GT1-motif	GGTTAAT	2	light responsive element

Table S3 Primer information used in this study

PCR primers for the recombinant plasmid (5'-3')		
qRT-StPUB51	Forward	TCAATCGTGGGTTCGAAGCA
	Reverse	CCCAGTCATTCCGTTTCCGA
qRT-ef1α	Forward	ATTGGAAACGGATATGCTCCA
	Reverse	TCCTTACCTGAACGCCTGTCA
pEGFP-StPUB51	Forward	ACGGGGGACGAGCTCGGTACCATGGCATCAATCGTGGGTTC
	Reverse	GCTCACCATGTCGACTCTAGACTGTATCCTGTATGCCCAGCCA
pRI201-StPUB51	Forward	TCTTCACTGTTGATACATATGATGGCATCAATCGTGGGTTC
	Reverse	TGTTGATTCAGAATTGTCGACTTACTGTATCCTGTATGCCC
pCPB121-StPUB51	I	GATCATTCCGTTTCCGATCACATTCTCTCTTTTGTATTCC
	II	GAATGTGATCGGAAACGGAATGATCAAAGAGAATCAATGA
	III	GAATATGATCGGAAAGGGAATGTTCACAGGTCGTGATATG
	IV	GAACATTCCCTTTCCGATCATATTCTACATATATATTCCT
	A	CTGCAAGGCGATTAAGTTGGGTAAC
	B	GCGGATAACAATTTCACACAGGAAACAG
NPTII	Forward	GCTATGACTGGGCACAACAG
	Reverse	ATACCGTAAAGCACGAGGAA
pGBKT7-StPUB51	Forward	TCAGAGGAGGACCTGCATATGATGGCATCAATCGTGGGTTC
	Reverse	ATGCGGCCGCTGCAGGTCGACTTACTGTATCCTGTATGCCCAGCC
pGADT7-SKP2A	Forward	GTACCAGATTACGCTCATATGATGTTAGGGGTAGAAGATTTGAGCTT
	Reverse	CAGCTCGAGCTCGATGGATCCTCAGTGAGCTGGATGGAGAACA
pGADT7-StGATA1	Forward	GTACCAGATTACGCTCATATGATGAAAATGGAAGCTATGTACTCAAAA
	Reverse	CAGCTCGAGCTCGATGGATCCTTAAATCCTCTGCTTTCTCATCTCC
pSPYCE-StPUB51	Forward	GAGAACACGGGGGACTCTAGAATGGCATCAATCGTGGGTTC
	Reverse	GTACATCCCGGGAGCGGTACCCTGTATCCTGTATGCCCAGCCA
pSPYNE-StSKP2A	Forward	GAGAACACGGGGGACTCTAGAATGTTAGGGGTAGAAGATTTGAGCTT
	Reverse	CTCCATCCCGGGAGCGGTACCGTGAGCTGGATGGAGAACACG
pSPYNE-StGATA1	Forward	GAGAACACGGGGGACTCTAGAATGAAAATGGAAGCTATGTACTCAAAA
	Reverse	CTCCATCCCGGGAGCGGTACCAATCCTCTGCTTTCTCATCTCCAA
